# Education Research: Educational Outcomes Associated With the Introduction of a Neurohospitalist Program

**DOI:** 10.1212/NE9.0000000000200131

**Published:** 2024-05-09

**Authors:** Shefali Dujari, Brian J. Scott, Carl A. Gold, Yingjie Weng, Kathryn A. Kvam

**Affiliations:** From the Department of Neurology & Neurological Sciences (S.D., B.J.S., C.A.G., K.A.K.), and Quantitative Sciences Unit (Y.W.), Stanford University, CA.

## Abstract

**Background and Objectives:**

As the prevalence of the neurohospitalist (NH) practice model grows, understanding its effect on trainee education is imperative. We sought to determine the impact of an academic NH program on neurology resident evaluations of faculty teaching.

**Methods:**

We performed a retrospective study of faculty teaching evaluations before and after the implementation of a full-time NH service. Primary outcomes were neurology resident evaluations of faculty teaching, which were compared in the pre-NH period (August 2010–July 2014) vs the post-NH period (August 2016–July 2018). In a secondary analysis, we used the difference-in-difference approach to analyze the effect of introducing the NH service on resident evaluation of faculty teaching compared with stroke and neurocritical care faculty controls. We performed an additional descriptive analysis of medical student evaluation of faculty teaching and described Residency In-service Training Exam scores and Accreditation Council for Graduate Medical Education (ACGME) resident survey data before and after the intervention.

**Results:**

There were 368 resident and 360 medical student evaluations of faculty teaching during the study period. Compared to the pre-NH period, the post-NH period had significantly higher resident evaluations of faculty teaching in 19 of 27 questions of faculty teaching, across 5 of the 6 ACGME core competencies. Within the competencies of patient care, practice-based learning and improvement, and systems-based practice, the NH teaching faculty were rated significantly higher across all questions. In the difference-in-difference model, resident evaluations of faculty teaching following the implementation of the NH service remained significantly improved compared with controls in teaching evidence-based medicine, teaching diagnostic algorithms, and explaining rationale for clinical decisions. Medical student ratings of faculty teaching were unchanged in the pre-NH and the post-NH period.

**Discussion:**

Neurology residents may benefit from the clinical expertise of NHs and their ability to teach evidence-based practice and role model systems-based practice. Given the central role NHs may play in trainee education, additional focus on both the local and national levels should be dedicated to further developing the teaching skills of NHs.

## Introduction

Neurohospitalists (NHs), physicians who subspecialize in the management of inpatients with neurologic disorders, are poised to enhance the clinical education of trainees.^1^ In a survey of residency program directors and department chairs, perceived advantages of the NH model on resident education included inpatient expertise, improved efficiency, and better availability.^2^ Several studies have demonstrated increased trainee satisfaction with hospitalist models in internal medicine and pediatrics compared with models with rotating outpatient physicians staffing inpatient services.^3^

To our knowledge, only one study has assessed the effect of NHs on trainee education.^4^ That study demonstrated no change associated with the implementation of a NH program on either resident or medical student ratings of faculty teaching.

At our institution, we fully transitioned to a NH model for general inpatient neurology staffing in 2016. In a previously published study on the clinical outcomes of the NH model at our institution, the NH model was associated with a significant reduction in risk-adjusted mean length of stay despite the increased patient complexity in the post-NH period.^5^ Secondary outcomes including mortality, in-hospital complication rate, cost, and patient satisfaction all remained stable. Educational outcomes for medical students and residents were not evaluated.

As the prevalence of the NH practice model grows, understanding its effect on trainee education is imperative to continue to prioritize and foster the growth of the next generation of neurologists. In this study, we sought to measure primarily the impact of the NH model on neurology resident evaluation of faculty teaching and secondarily the effect on medical students, performance on the Residency In-Service Training Examination (RITE), and resident perceptions of patient safety and teamwork as assessed on the annual Accreditation Council for Graduate Medical Education (ACGME) survey.

## Methods

### Study Design

We conducted a retrospective analysis of resident evaluations of neurology teaching faculty before and after the implementation of a NH model. A secondary analysis of the resident evaluations using a difference-in-difference (DID) design with separate stroke and neurocritical care services serving as controls was additionally conducted. We chose these 2 inpatient services at the same academic hospital as controls because they were most similar to the general neurology service in patient population and teaching structure. Additional descriptive analysis of medical student evaluation of faculty teaching and exploratory analyses of RITE scores and ACGME resident survey data were also performed.

### Background and Setting

As previously described, the general neurology inpatient service at our institution was staffed by a pool of 24–26 faculty before the introduction of the NH model.^5^ In this “traditional model,” each faculty member would attend on-service for 1–3 weeks per year. The on-service attending responsibilities included serving as the primary attending for the general neurology inpatient ward service and the general neurology consult service. Depending on their subspecialty, the on-service attending would also maintain some of their outpatient clinics and/or staff on the inpatient stroke or inpatient epilepsy services during this time.

### Intervention

After a 2-year pilot period with 1–2 NHs and inpatient staffing split between NH and non-NH faculty, by August 1, 2016, our institution fully restructured the general neurology inpatient service to be staffed by 3–4 NH attendings 98% of the year (51/52 weeks). Each NH attending was on-service for at least 8 weeks per year. The NH attending was responsible for the general neurology ward service and the general neurology consult service (collectively referred to as NH services in this manuscript), with no additional required clinical responsibilities. After rounds, the on-service NH attending was available to attend case management rounds, staff new admissions and consultations, and attend to other patient care needs with the trainees. In addition to the attending, the neurology ward service consisted of a senior neurology resident (postgraduate year [PGY]-4), a junior neurology resident (PGY2), and a third-year medical student completing their core neurology rotation. The neurology consult service included 1 neurology resident (PGY2 or PGY3) and, at times, a fourth-year medical student completing an advanced elective (subinternship). In addition, there was an evening “swing” resident (PGY2) and a night float resident (PGY2 or PGY3) who would occasionally staff patients in person or by phone with the attending. The on-service NH led a weekly orientation and clinical goal-setting meeting with the rounding team and provided both real-time and weekly summative individualized feedback for each trainee. In the post-NH model, given the consistent presence of the NH division on the general neurology inpatient services, each attending spent on average 1–3 weeks per year across all 3 years working with each resident.

### Control Group

The stroke and neurocritical care services served as controls in the DID model for resident evaluations. Similar to the general neurology inpatient ward service and the general neurology inpatient consult service, neurology residents rotated on the stroke and neurocritical care services in 2-week to 4-week blocks and had daily teaching rounds with the on-service attending. During the study period, residents cared for both primary and consult patients on the stroke service and consult patients on the neurocritical care service. The team structure of the stroke and neurocritical care services included 1–2 junior residents (PGY2 and/or PGY3). While there was no senior resident (as was present on the general neurology ward service), each service always had a dedicated vascular or neurocritical care fellow supporting and overseeing the residents.

### Study Population

Our study cohort included residents' and medical students' evaluations of faculty teaching from August 2010 to July 2018. The pre-NH period was defined as evaluations issued between August 2010 and July 2014. The post-NH period was from August 2016 to July 2018. We excluded the August 2014–July 2016 pilot period in our primary and secondary analyses.

Neurology residents and medical students were anonymously identified based on dates of completed evaluations of faculty teaching. The listed inpatient rotation dates on the evaluation were matched to the attending rounding schedules. We restricted resident evaluations to those that included an inpatient resident rotation title (“ward senior,” “ward junior,” “consult resident,” etc.) for the service to ensure that no fellow evaluations or outpatient evaluations were included. This was most relevant in the pre-NH period when attendings that were primarily working with trainees in the outpatient setting also staffed the inpatient neurology service.

### Outcomes

Faculty evaluations were distributed electronically to residents and medical students at the end of every rotation block by the respective program coordinator. Residents completed standardized evaluations of clinical faculty across the 6 ACGME core competencies^6^ (patient care, medical knowledge, professionalism, systems-based practice, practice-based learning, and interpersonal and communication skills) and on overall clinical excellence (eFile 1). Responses were on a bidirectional Likert scale (1–6: “strongly disagree,” “disagree moderately,” “disagree slightly,” “agree slightly,” “agree moderately,” and “strongly agree”) with the exception of “overall clinical excellence,” which had ratings 1–9 (1–3: “below expectations;” 4–6: “meets expectations;” and 7–9: “exceeds expectations”) and were submitted anonymously through an electronic portal.

Medical students evaluated faculty on learning climate, feedback, facilitation of learning, professionalism, and overall quality of instruction based on a Likert scale (1–5: “poor,” “fair,” “good,” “very good,” and “excellent”; eFile 2).

### Statistical Analysis

#### Residents

We reported the mean and standard deviations of the Likert scales of residents' evaluations for the pre-NH and post-NH groups. The high baseline scores on the ordinal Likert scale in the pre-NH period limited the ability to distinguish change using median scores; therefore, for the primary analysis, mean scores were used. The median scores of residents' evaluations are reported separately in the supplement. To compare the difference between the pre-NH and post-NH groups, we applied linear regression models to the period (post-NH vs pre-NH) on each of the Likert scales and reported the point estimates (β coefficients) and the corresponding uncertainties (95% confidence intervals). Coefficients from models were interpreted as the mean differences of the Likert scales in the post-NH group compared with the pre-NH group. We adjusted family-wise type I error for multiple comparisons using the Benjamini method that controls for the false discovery rate (FDR).^7^ Adjusted *p* < 0.05 was interpreted as statistically significant.

A secondary analysis of resident evaluations of teaching was done using the DID approach.^8-10^ In this model, the change in the outcome over time in the intervention and control groups is compared. The DID model is an important analytical approach when randomization is not possible as it allows for control of other factors outside of the intervention that may impact the outcome of interest over time, for example, potential temporal changes in the institution-wide learning environment and evaluation patterns among trainees. In our model, resident evaluations of stroke and neurocritical care faculty teaching in the corresponding pre-NH and post-NH periods served as controls.

In this analysis, for each of the Likert-scale outcomes, we included the period (post-NH vs pre-NH), the group (residents rotating on the NH services vs stroke and neurocritical care services), and the interaction between the 2 (DID estimator) in the linear regression model. The impact of the NH program would be represented by the DID estimators, which was interpreted as the difference in the changes of the Likert scales from the pre-NH to the post-NH period for resident rating of faculty teaching on the NH services compared with the resident ratings of stroke and neurocritical care faculty teaching. The coefficient estimates for the DIDs along with the corresponding 95% confidence intervals were reported.

We additionally describe changes in RITE scores and ACGME resident survey data after the NH intervention. For the RITE data, we report the change in the average total score and the percentile rank, as well as the change in the average “clinical adult” section score and the percentile rank in the post-NH (2017, 2018) vs pre-NH (2012, 2013, 2014) periods. For the ACGME resident survey data, we selected questions within the category of teamwork and patient safety felt to be reflective of NH practice, specifically “culture reinforces patient safety responsibility,” “participated in quality improvement,” “work in interprofessional teams,” and “effectively work in interprofessional teams.” These were answered on 5-point Likert scales. We report the change in the average program mean in the post-NH (2017, 2018) vs pre-NH (2012, 2013, 2014) period. Data limitations prevented more detailed statistical inference; specifically, data were not available for the entire pre-NH period and only aggregate scores were available for both the RITE scores and ACGME survey data by year. Without estimates of the variabilities and standard deviations of the scores for the RITE and ACGME survey data, further descriptive analysis could not be performed.

#### Medical Students

We described the distributions of responses from the medical students' evaluations by the pre-NH and the post-NH group and compared the differences using the standardized mean difference (SMD), which is the mean difference between the 2 groups divided by the pooled standard deviation. The higher the SMD, the greater the magnitude of differences between the 2 groups. Cohen offered the following guidelines for interpreting the magnitude of the SMD: small, SMD = 0.2; medium, SMD = 0.5; and large, SMD = 0.8.^11,12^

All analyses were performed using R statistical programming languages, version 3.4.3.^13^

### Standard Protocol Approvals, Registrations, and Patient Consents

This study was approved by the Stanford University Institutional Review Board.

### Data Availability

Anonymized data not published within the article are available at the request of other investigators for the purposes of replicating procedures and results. Qualified investigators interested in obtaining data access may contact the corresponding author (S.D.).

## Results

### Residents

Demographic data of teaching faculty evaluations in the pre-NH and post-NH periods are included in Table 1. There were 368 resident evaluations of faculty teaching (227 preintervention and 141 postintervention) over the study period. Owing to the retrospective nature of the study, we were unable to quantify the evaluation response rate. We estimated the number of evaluations distributed based on the number of residents and attendings on-service during a rotation block (eTable 1). Using this estimate, the number of evaluations received over an estimated number of evaluations distributed was 46% in the pre-NH cohort (227/489) and 35% in the post-NH cohort (141/400).

**Table 1:** Faculty Demographics of Included Evaluations

	Intervention		Control	
	Pre	Post	Pre	Post
Total evaluations	227	141	116	138
Evaluations of female faculty, n (%)	84 (37)	55 (39)	66 (57)	80 (58)
Years since residency graduation of evaluated faculty, median (IQR)	16 (7–24)	4 (2–5)	8 (7–10)	12 (4–14)

Abbreviation: IQR = interquartile range.

The NH intervention was associated with significantly higher resident ratings of faculty teaching in most survey items post-NH as compared with the pre-NH period (Table 2). A significant impact of the NH program was seen in 19 of 27 questions regarding faculty teaching, including overall clinical excellence, and spanning across 5 of the 6 ACGME core competencies—patient care, medical knowledge, practice-based learning and improvement, interpersonal and communication skills, and systems-based practice. The largest improvements were noted in teaching pathophysiology (difference 0.46, 95% CI 0.17–0.76, FDR-adjusted *p* < 0.01), cost-benefit decision making (0.41, 95% CI 0.26–0.55, *p* < 0.01), how to use resources in the system (0.34, 95% CI 0.21–0.48, *p* < 0.01), scientific evidence (0.31, 95% CI 0.05–0.57, *p* = 0.03), and treatment algorithms (0.27, 95% CI 0.13–0.41, *p* < 0.01). Overall clinical excellence of faculty was rated significantly higher in the post-NH period (0.46, 95% CI 0.22–0.69, *p* < 0.01).

**Table 2:** Resident Rating of General Neurology Service Faculty Teaching Before and After Introduction of a Neurohospitalist Service

Questions, mean (SD)	Pre-NH	Post-NH	Difference (95% CI)	*p* Value^c^
No. of evaluations	227	141		
Overall clinical excellence^a^	7.80 (1.29)	8.25 (0.80)	0.46 (0.22 to 0.69)	**<0.01**
Systems-based practice^b^				
Role models working in interprofessional teams	5.63 (0.73)	5.89 (0.31)	0.27 (0.14 to 0.39)	**<0.01**
Teaches how to use resources in the system	5.50 (0.78)	5.84 (0.37)	0.34 (0.21 to 0.48)	**<0.01**
Teaches cost-benefit decision making	5.41 (0.81)	5.82 (0.40)	0.41 (0.26 to 0.55)	**<0.01**
Medical knowledge^b^				
Record of scholarly activities	5.81 (0.50)	5.78 (0.45)	−0.02 (−0.18 to 0.13)	0.75
Creates an environment of scholarly inquiry	5.81 (0.54)	5.85 (0.36)	0.04 (−0.10 to 0.18)	0.58
Teaching adds to my medical knowledge	5.70 (0.67)	5.87 (0.34)	0.17 (0.06 to 0.29)	**<0.01**
Teaches when to order tests	5.61 (0.69)	5.85 (0.36)	0.24 (0.12 to 0.36)	**<0.01**
Teaches evidence-based medicine	5.42 (1.34)	5.73 (1.03)	0.26 (0.13 to 0.38)	**<0.01**
Teaches pathophysiology	5.23 (1.59)	5.70 (0.94)	0.46 (0.17 to 0.76)	**<0.01**
Patient care^b^				
Explains rationale for clinical decisions	5.74 (0.62)	5.94 (0.25)	0.19 (0.08 to 0.30)	**<0.01**
Teaches diagnostic algorithms	5.65 (0.67)	5.89 (0.31)	0.24 (0.12 to 0.36)	**<0.01**
Teaches treatment algorithms	5.42 (1.33)	5.72 (1.04)	0.30 (0.04 to 0.56)	**0.03**
Teaches scientific evidence	5.62 (0.70)	5.88 (0.35)	0.31 (0.05 to 0.57)	**0.03**
Practice-based learning and improvement^b^				
Asks about differential diagnosis	5.70 (0.64)	5.88 (0.33)	0.17 (0.06 to 0.29)	**<0.01**
Actively teaches trainees in clinical settings	5.70 (0.69)	5.91 (0.28)	0.21 (0.09 to 0.33)	**<0.01**
Teaches when to consider consults	5.66 (0.68)	5.86 (0.34)	0.20 (0.08 to 0.32)	**<0.01**
Interpersonal and communication skills^b^				
Stimulates critical thinking	5.72 (0.68)	5.86 (0.34)	0.15 (0.02 to 0.27)	**0.03**
Encourages questions	5.77 (0.64)	5.91 (0.29)	0.14 (0.02 to 0.25)	**0.03**
Teaches at the appropriate level	5.78 (0.63)	5.89 (0.33)	0.11 (0 to 0.23)	0.07
Motivates learners to expand knowledge	5.74 (0.64)	5.91 (0.29)	0.17 (0.06 to 0.28)	**<0.01**
Provides specific feedback	5.55 (0.82)	5.76 (0.52)	0.20 (0.05 to 0.36)	**0.02**
Professionalism^b^				
Does not belittle or humiliate learners	5.84 (0.60)	5.91 (0.32)	0.07 (−0.04 to 0.17)	0.26
Demonstrates respect for trainees	5.84 (0.57)	5.91 (0.32)	0.07 (−0.04 to 0.17)	0.25
Teaches professional behavior	5.86 (0.55)	5.92 (0.30)	0.06 (−0.04 to 0.16)	0.25
Exhibits professional behavior in patient care	5.85 (0.57)	5.92 (0.30)	0.08 (−0.03 to 0.18)	0.19
Role models professionalism	5.84 (0.60)	5.93 (0.26)	0.09 (−0.02 to 0.19)	0.14

Abbreviation: NH = neurohospitalist.

a Likert scale 1–9 (1–3: below expectations; 4–6: meets expectations; and 7–9: exceeds expectation).

b Likert scale 1–6 (1 = strongly disagree; 2 = disagree moderately; 3 = disagree slightly; 4 = agree slightly; 5 = agree moderately; and 6 = strongly agree).

c *p* Values were adjusted for family-wise type I error using the Benjamini method that controls for the false discovery rate to determine statistical significance. Adjusted *p* value <0.05 was considered as statistically significant.

In the DID analysis, using resident evaluations of stroke and neurocritical care faculty teaching as a control, the positive impact of the NH model on resident evaluations of teaching was still significant in 3 of the 27 questions (Table 3, Figure). Compared with controls, statistically significant improvement with the NH model was noted in teaching evidence-based medicine (0.29, 95% CI 0.12–0.47, *p* = 0.02), teaching diagnostic algorithms (0.28, 95% CI 0.10–0.45, *p* = 0.02), and explaining rationale for clinical decisions (0.26, 95% CI 0.09–0.43, *p* = 0.02).

**Table 3:** Difference-in-Difference Analysis of Resident Rating of General Neurology Service Faculty Teaching Before and After Introduction of a Neurohospitalist Service

Questions	Difference^a^ (95% CI)	*p* Value
Overall clinical excellence	0.44 (0.08 to 0.80)	0.09
Systems-based practice		
Role models working in interprofessional teams	0.14 (−0.05 to 0.33)	0.24
Teaches how to use resources in the system	0.21 (0 to 0.42)	0.16
Teaches cost-benefit decision making	0.25 (0.02 to 0.48)	0.12
Medical knowledge		
Record of scholarly activities	0.01 (−0.26 to 0.28)	0.95
Creates an environment of scholarly inquiry	0.07 (−0.20 to 0.34)	0.70
Teaching adds to my medical knowledge	0.12 (−0.05 to 0.30)	0.26
Teaches when to order tests	0.24 (0.06 to 0.42)	0.06
Teaches evidence-based medicine	0.29 (0.12 to 0.47)	**0.02**
Teaches pathophysiology	0.43 (0.03 to 0.82)	0.12
Patient care		
Explains rationale for clinical decisions	0.26 (0.09 to 0.43)	**0.02**
Teaches diagnostic algorithms	0.28 (0.10 to 0.45)	**0.02**
Teaches treatment algorithms	0.31 (−0.05 to 0.67)	0.20
Teaches scientific evidence	0.34 (0.03 to 0.66)	0.12
Practice-based learning and improvement		
Asks about differential diagnosis	0.09 (−0.09 to 0.27)	0.41
Actively teaches trainees in clinical settings	0.16 (−0.02 to 0.33)	0.20
Teaches when to consider consults	0.16 (−0.03 to 0.35)	0.20
Interpersonal and communication skills		
Stimulates critical thinking	0.03 (−0.15 to 0.20)	0.80
Encourages questions	0.03 (−0.14 to 0.20)	0.77
Teaches at the appropriate level	0.04 (−0.13 to 0.21)	0.73
Motivates learners to expand knowledge	0.09 (−0.08 to 0.25)	0.41
Provides specific feedback	0.13 (−0.11 to 0.37)	0.34
Professionalism		
Does not belittle or humiliate learners	0.07 (−0.08 to 0.22)	0.41
Demonstrates respect for trainees	0.09 (−0.06 to 0.25)	0.37
Teaches professional behavior	0.10 (−0.03 to 0.24)	0.24
Exhibits professional behavior in patient care	0.12 (−0.02 to 0.26)	0.20
Role models professionalism	0.14 (0 to 0.29)	0.16

Abbreviation: NH = neurohospitalist.

a Where the difference is equal to (post-NH intervention group mean – post-NH control group mean) − (pre-NH intervention group mean – pre-NH control group mean), for example, for “overall clinical excellence”: (8.25 − 7.83) − (7.80 − 7.82) = 0.44. A difference >0 indicates an incremental increase in the rating of faculty teaching in the intervention group relative to the control group over time. A difference <0 indicates a decrease in faculty rating in the intervention group relative to the control group over time.

Adjusted *p* value <0.05 was considered as statistically significant.

**Figure:**
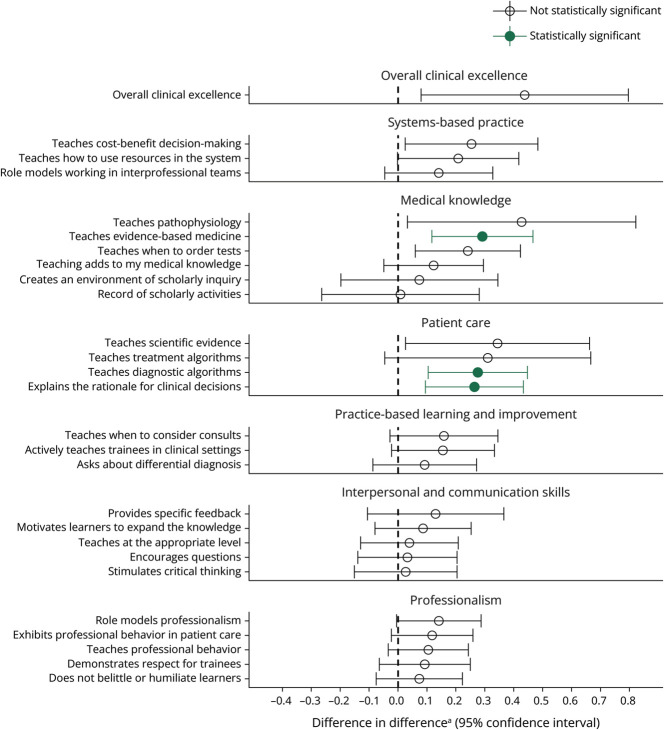
Difference-in-Difference Analysis of Resident Rating of General Neurology Service Faculty Teaching Before and After Introduction of a Neurohospitalist Service ^a^Where the difference is equal to (post-NH intervention group mean − post-NH control group mean) − (pre-NH intervention group mean − pre-NH control group mean). NH = neurohospitalist.

With regard to resident RITE scores, the percentage of total questions correct increased by 4% and the total percentile rank compared with programs nationally increased by 21 in the post-NH period in comparison with the pre-NH period. Within the clinical adult section of the RITE, the percent correct increased by 1% and the percentile rank increased by 9. For ACGME survey data, on a 5-point Likert scale, “culture reinforces patient safety responsibility” increased by 0.26 points, “participated in quality improvement” improved by 1.29 points, “work in interprofessional teams” increased by 0.27 points, and “effectively work in interprofessional teams” increased by 0.25 points in the post-NH period in comparison with the pre-NH period. Unfortunately, limitations in the available data prevent statistical analysis on RITE scores and ACGME survey data, as described in the Methods section.

### Medical Students

There were 360 medical student evaluations of faculty teaching (210 pre- and 150 postintervention). Using the SMD, medical student ratings of NH faculty teaching did not demonstrate a significant change between the pre-NH and post-NH periods (Table 4).

**Table 4:** Medical Student Evaluation of Faculty Teaching

Questions,^a^ mean (SD)	Pre-NH	Post-NH	SMD^b^
No. of evaluations	210	150	
Learning climate	4.7 (0.67)	4.7 (0.72)	0.02
Feedback	4.3 (0.98)	4.5 (0.98)	0.13
Facilitation of learning	4.6 (0.73)	4.7 (0.71)	0.05
Professionalism	4.8 (0.62)	4.8 (0.56)	0.13
Overall quality of instruction	4.6 (0.75)	4.6 (0.74)	0.07

Abbreviations: NH = neurohospitalist; SMD = standardized mean difference.

a Likert-scale responses 1–5: “poor,” “fair,” “good,” “very good,” and “excellent.”

b Suggested guidelines for interpreting the magnitude of the SMD: small, SMD = 0.2; medium, SMD = 0.5; and large, SMD = 0.8.

## Discussion

In this study, the implementation of the NH program was associated with significantly improved resident ratings of faculty teaching in 19 of 27 questions, spanning 5 of the 6 ACGME competencies. Notably, the intervention was associated with significantly higher ratings across all questions in the competencies of patient care, practice-based learning and improvement, and systems-based practice. There was no significant difference found in the questions addressing the competency of professionalism, which is likely related to high scores within this domain both before and after the implementation of the NH intervention. When using resident evaluations of stroke and neurocritical care faculty teaching as a control, this significant incremental impact of the NH service was still evident in teaching evidence-based medicine, explaining rationale for clinical decisions, and teaching diagnostic algorithms. The remaining questions were unchanged between the pre-NH and post-NH periods, despite a significant decrease in mean adjusted length of stay and higher acuity of illness in the patient population in the post-NH period, as described in a separate study performed at our institution.^5^ The field of NH medicine was largely born out of financial and regulatory pressures to provide cost-effective, safe, and timely care in the inpatient setting.^14^ The findings of our study highlight the specific advantages of the NH model and skillset of NH faculty as educators of evidence-based, high-quality inpatient care.

Several studies have demonstrated the benefit of internal medicine and pediatric hospitalists on trainee education, including improved rating of teaching effectiveness, knowledge of inpatient medicine and pathophysiology, evidence-based medicine, and feedback.^3,15-19^ Only one other study evaluating educational outcomes associated with the implementation of a NH program has been published.^4^ In that study, the implementation of a NH program was associated with stable resident evaluations of attendings and a nonsignificant trend toward improved medical student evaluations of attendings in overall teaching skills, approachability, cultural sensitivity, and conveyance of information after the creation of the NH program. We postulate that the lack of a positive impact on trainee evaluations may in part be related to the period studied, as the post-NH period included a period of transition, with less than 40% of the service time covered by a NH attending. In our study, as the transition time was not included, 98% of the service time in the post-NH period was staffed by a NH attending.

The main perceived disadvantages of the hospitalist model on trainee education in both internal medicine^20^ and neurology^2^ are decreased exposure to subspecialty attendings. Opportunities to mitigate and even enhance subspecialty exposure through the flexibility of a NH program that have been previously highlighted include subspecialty-focused morning reports, journal clubs, and didactics, social events with subspecialty faculty, early elective time, and subspecialty rotations in residency.^21^ In addition, on our services, we have active engagement with subspecialty consultants such as neuromuscular, neuroimmunology, and neuro-oncology consultants through inpatient consults and case conference discussions on complex hospitalized patients, which further enhances trainee exposure to subspecialty attendings.

Our residency program strives to achieve a balance between inpatient and outpatient clinical exposure. Adult neurology residents who graduated in the final year of the study period (June 2018) spent approximately 13 months on clinic or elective rotations and 10.5 months on rotations with both inpatient and outpatient exposure during their training. Striking an appropriate split between inpatient and outpatient attending exposure is an important consideration when transitioning an academic inpatient neurology service to the NH model given the potential influence on fellowship and subsequent career choices.^22^

Medical student evaluations of faculty teaching were unchanged between the pre-NH and post-NH periods. We speculate this discrepancy as compared with resident ratings relates to the broader and deeper impact the NH model has on resident education, allowing NH faculty to work with residents periodically throughout residency and enabling more targeted teaching to help individual residents advance across the milestones. Interestingly, it has previously been noted that medical students' satisfaction scores are more closely linked to satisfaction with resident teaching rather than attending teaching.^19^ In addition, it has been shown that medical students value different qualities in an educator than residents; whereas residents prioritize efficient teaching skills such as stimulation of learning and problem-solving, students most appreciate creating a caring and supportive learning environment.^23^ Our faculty scored highly in these areas both before and after the introduction of a NH service. Previous studies have called for more evaluation of the impact of hospitalist models of medical student teaching,^24^ but few have been performed.^15,16,19,25^ Similar to resident outcomes, these limited studies have demonstrated a positive impact on medical student evaluations of faculty with the introduction of internal medicine and pediatric hospitalists.

Our study highlights the central role academic NHs can play as clinical teachers. We posit this is in part related to the concerted effort our NH faculty invests in weekly clinical goal-setting with learners and real-time and summative feedback over the course of their inpatient rotations during training.^26-29^ In internal medicine, increased faculty presence in the afternoon and/or evening has been associated with higher resident satisfaction and higher ratings of education.^30^ Our faculty have all engaged in optional University-sponsored faculty development courses related to medical education. Importantly, the NH model allows for a unique, longitudinal mentoring relationship with residents over the course of their training, which can appropriately evolve as trainees progress from junior to senior residents.^31^ In addition to on-service teaching, our faculty are greatly involved in resident development, such as leading a longitudinal resident quality improvement curriculum, leading a longitudinal communication coaching program, leading a resident well-being program, and serving as individual mentors for resident-led research projects.

In our unique DID analysis using stroke and neurocritical care faculty as controls, residents’ rating of teaching evidence-based medicine, explaining rationale for clinical decisions, and teaching diagnostic algorithms was significantly higher in the post-NH period. In a qualitative study of pediatric hospitalists as educators, the exemplary hospitalist educator possessed teaching skills, which specifically balanced patient care and efficiency with education and learner needs, focusing on a results-oriented thought process and clinical reasoning, as well as patient care skills including knowledge acquisition and staying up-to-date on current evidence and literature.^23^ This demonstrates the value NHs can bring to trainee education in these specific domains and highlights teaching skills that should be emphasized in NH training programs.

This study also emphasizes the strengths of the NH model on education within the specific domains of patient care, practice-based learning and improvement, and systems-based practice. In addition to higher ratings of faculty teaching within these competencies, the ACGME resident survey data demonstrate increased resident engagement in patient safety, quality improvement, and interprofessional teamwork in the post-NH period. This highlights the unique advantages of a NH, including timely delivery of complex, acute neurologic care, advocating for patient safety, and advancement of high-quality inpatient neurologic care.^14^ Studies of pediatric and medicine hospitalists have similarly demonstrated higher ratings of hospitalists for teaching effectiveness, emphasis on evidence-based care with specific knowledge of inpatient medicine, and emphasis on cost-effectiveness.^3,19^ This is postulated to reflect the specialized expertise the hospitalist has in the inpatient setting by virtue of extensive time spent and, for many, specific training done in this setting. For example, during the post-NH study period, each NH faculty member had completed either a NH or stroke fellowship.

The current study has several potential limitations. First, it is important to note that although trainee evaluations of faculty are useful as a feedback mechanism to clinical educators^32^ and are relied upon in the academic promotion process, these types of evaluations have several potential flaws. The halo effect, ceiling effect, and gender bias may impact the validity of these evaluations.^33,34^ For example, female faculty, particularly in subspecialties with lower relative female representation such as neurology, have significantly lower median evaluation scores in comparison with males.^35^ The percentage of evaluations for female faculty in our cohort was similar in the pre-NH and post-NH periods but lower in the intervention group (NH) than in the control group (Table 1), which may have influenced the results of the DID analysis. We also found that faculty in the post-NH period were fewer years out of training (4 years) than in the pre-NH period (16 years). There is limited research in graduate medical education on how faculty age and/or rank influences trainee evaluations; available research suggests a range of moderately higher ratings of faculty closer to their own training to no effect of faculty rank on trainee rating.^36,37^ Additional variables that were not collected but may have contributed to bias in the evaluations include faculty race, trainee gender, race, and years in training, and concordance between faculty and trainee gender and race.^38,39^ Based on the high pre-NH mean scores, it is also likely that the ceiling effect limited our ability to detect a more significant change.

Second, given the study design, we were unable to determine the resident evaluation response rate; however, we estimate a response rate of 46% in the pre-NH period and 35% in the post-NH period. Other similar studies of medicine hospitalists' impact on resident education report response rates of 53%–91%.^17-19^ At our institution, 1–2 days before the end of a block, residents submit the names of the faculty members they worked with during the rotation to the residency administrators who then generate an evaluation form, which is sent to the resident. On rotations such as swing and night float where residents do not work with 1 primary attending on daily rounds, they may be less likely to submit faculty names. Therefore, we suspect the denominator in our calculations likely overestimates the actual number of evaluations sent out. In addition, we note that there is a small decrease in the estimated response rate between the pre-NH and post-NH periods. We postulate that given the smaller volume of attendings on the NH services in the post-NH period, residents may have been less likely to fill out several evaluations for the same faculty member across several rotations.

Third, there may not be sufficient statistical power to detect meaningful differences in outcomes with low event rates or small sample sizes. This likely limited the ability to measure a difference in medical student evaluations, as these consist of 5 broad, thematic questions evaluated on a 5-point scale.

Finally, the primary outcomes of this study provide level 1 data (reaction) to support the NH model for trainee education in the Kirkpatrick model, a commonly used schema in education research to evaluate the degree of learning achieved by a training program.^40^ We describe the RITE (level 2) and ACGME survey data (level 3), although limitations in data availability prevented rigorous statistical analysis. In addition, other programmatic changes may have also contributed to the change in scores, particularly the RITE scores. Other outcomes to understand changes in behavior (level 3) and results (level 4) may have been informative, but were not captured, such as the neurology clinical evaluation exercise resident scores and patient outcomes. Beyond outcome measures, it is also important to understand the process by which trainees learn in educational settings,^41^ such as direct observation of faculty teaching or qualitative review of teaching feedback.

Our findings suggest that the NH model can offer unique educational value to trainees, particularly within the areas of teaching evidence-based medicine, explaining clinical decision making, and teaching diagnostic algorithms. Given the critical and growing role that NHs will play in resident and medical student education, additional emphasis should be placed on developing clinician educator skills during NH fellowship^42^ and as NH faculty through professional development courses and education-specific funding,^43^ on both a local and a national level.^44,45^

Future studies are needed to investigate what qualities and skills make for exemplary inpatient neurology teaching. While ward rounds represent a crucial part of the trainee experience, limited research has been conducted to understand how to optimize teaching in the inpatient setting, with trainees often reporting difficulty learning because of time constraints, workload, interruptions, and patient care needs.^46^ More research must be done in this area to understand the optimal format, content, and setting to inform faculty development needs for NHs working with trainees.

## Appendix Authors

**Table:**

Name	Location	Contribution
Shefali Dujari, MD	Stanford University, CA	Assisted in the study design; interpreted the data; drafted and edited the manuscript for intellectual content
Brian J. Scott, MD	Stanford University, CA	Assisted in the study design; interpreted the data; drafted and edited the manuscript for intellectual content
Carl A. Gold, MD, MS	Stanford University, CA	Assisted in the study design; interpreted the data; drafted and edited the manuscript for intellectual content
Yingjie Weng, MHS	Stanford University, CA	Assisted in the study design; major role in database management; performed all statistical analyses and interpreted the data; edited the manuscript for intellectual content
Kathryn A. Kvam, MD	Stanford University, CA	Designed and conceptualized the study; major role in data acquisition; interpreted the data; drafted and edited the manuscript for intellectual content

## Glossary

ACGME: Accreditation Council for Graduate Medical Education
DID: difference-in-difference
FDR: false discovery rate
NH: neurohospitalist
PGY: postgraduate year
RITE: Residency In-Service Training Examination
SMD: standardized mean difference
